# When imaging speaks louder than symptoms: a case report

**DOI:** 10.1093/ehjcr/ytag379

**Published:** 2026-05-20

**Authors:** Musa Nader, Kayan Lam, Tom Vromen, Sjoerd Bouwmeester, Erwin Tan

**Affiliations:** Department of Cardiothoracic Surgery, Catharina Hospital, 5602 ZA Eindhoven, Netherlands; Department of Cardiothoracic Surgery, Catharina Hospital, 5602 ZA Eindhoven, Netherlands; Department of Cardiology, Maxima Medisch Centrum, 5504 DB Eindhoven, Netherlands; Department of Cardiology, Catharina Hospital, 5602 ZA Eindhoven, Netherlands; Department of Cardiothoracic Surgery, Catharina Hospital, 5602 ZA Eindhoven, Netherlands

**Keywords:** Cutibacterium acnes, Prostetic valve endocarditis, Aortic valve dehiscence, Case report

## Abstract

**Background:**

Late prosthetic valve dehiscence is a rare but life-threatening complication, most commonly associated with infective endocarditis. Low-virulence organisms such as *Cutibacterium acnes* can cause indolent infections with minimal clinical signs, making diagnosis particularly challenging.

**Case summary:**

A 64-year-old man with a history of surgical aortic valve replacement with a mechanical prosthesis (2006) was admitted for elective VVI pacemaker implantation due to permanent atrial fibrillation with slow ventricular response and suspected intermittent complete atrioventricular block. During the procedure, fluoroscopy incidentally revealed abnormal mobility of the aortic prosthesis, raising suspicion for valve dehiscence. Transthoracic and transoesophageal echocardiography confirmed significant prosthetic rocking without relevant paravalvular regurgitation. Urgent surgical re-exploration demonstrated near circumferential dehiscence of the prosthesis. The valve was explanted and replaced with a bioprosthesis. Microbiological cultures of valve tissue and blood grew *Cutibacterium acnes*, consistent with chronic prosthetic valve endocarditis. The postoperative course was uneventful, and the patient was discharged on targeted antibiotic therapy. At 6-week follow-up, he remained asymptomatic with normal prosthetic valve function.

**Discussion:**

This case highlights a rare presentation of late mechanical prosthetic valve dehiscence due to indolent *Cutibacterium acnes* infection in the absence of overt clinical signs. It underscores the diagnostic value of incidental imaging findings and the need for a high index of suspicion for low-grade infective endocarditis in patients with prosthetic valves, even many years after implantation.

Learning points

- Rocking mechanical valve on fluoroscopy is an emergency red flag even without symptoms.
- This case underscores the importance of maintaining clinical vigilance and the potential role of incidental imaging in diagnosing life-threatening but clinically occult complications.

## Introduction

Prosthetic valve dehiscence is a rare but serious complication following surgical valve replacement, most frequently associated with infective endocarditis.^1^ While early prosthetic valve endocarditis typically presents with acute and overt clinical manifestations, late infections may follow a more indolent course and remain clinically silent for prolonged periods.^1,2^ This is particularly the case for low-virulence organisms such as *Cutibacterium acnes*, a slow-growing Gram-positive anaerobic bacterium that is part of the normal skin flora but increasingly recognized as a cause of prosthetic valve endocarditis.^3^

Due to its low pathogenicity, *C. acnes* infection is often characterized by subtle or absent systemic symptoms, low inflammatory markers, and negative initial blood cultures, frequently leading to delayed diagnosis. In such cases, imaging plays a pivotal role in detecting structural complications, including prosthetic valve dehiscence, which may occur even in the absence of significant paravalvular regurgitation or heart failure symptoms.

We report a case of late, near-circumferential dehiscence of a mechanical aortic valve prosthesis caused by *Cutibacterium acnes*, incidentally detected during an unrelated procedure. This case highlights the challenges in diagnosing indolent prosthetic valve endocarditis and underscores the importance of maintaining a high index of suspicion, even many years after valve implantation.

## Summary figure

**Figure:**
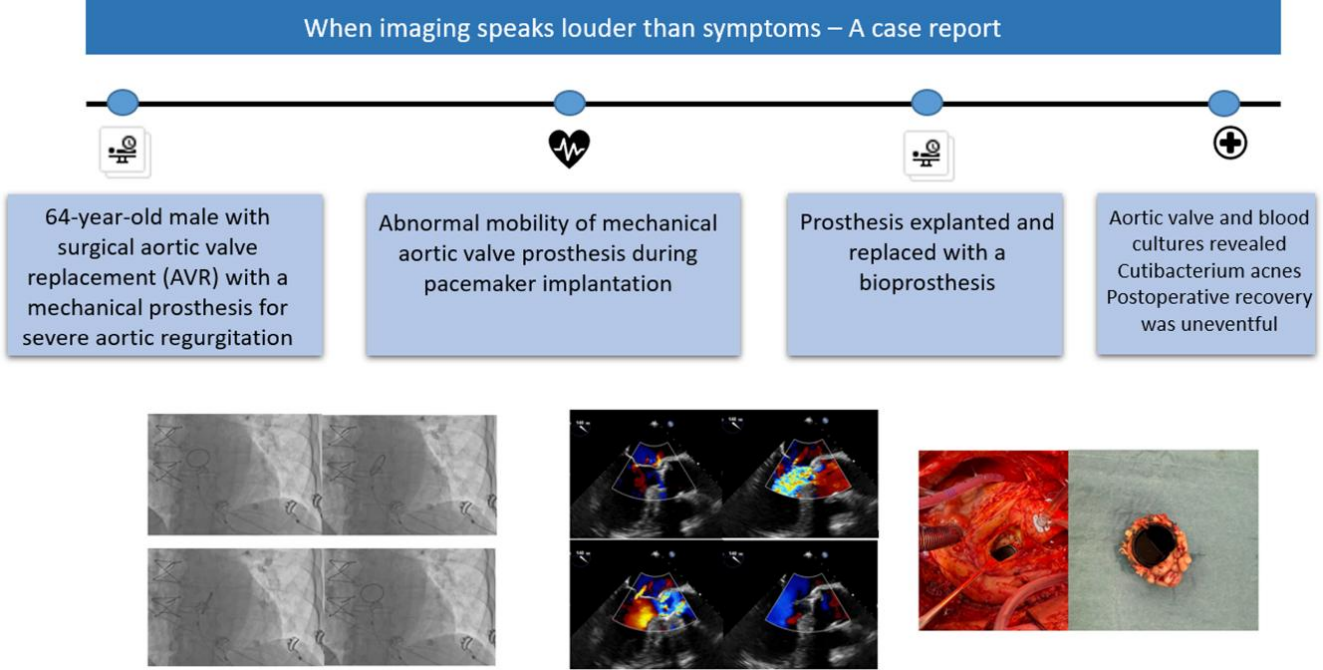


## Case presentation

A 64-year-old male with a medical history of surgical aortic valve replacement (AVR) in 2006 with a St. Jude mechanical prosthesis for severe aortic regurgitation was admitted for elective implantation of a VVI pacemaker. The indication was permanent atrial fibrillation with a slow ventricular response and presumed intermittent complete atrioventricular block.

**Figure 1:**
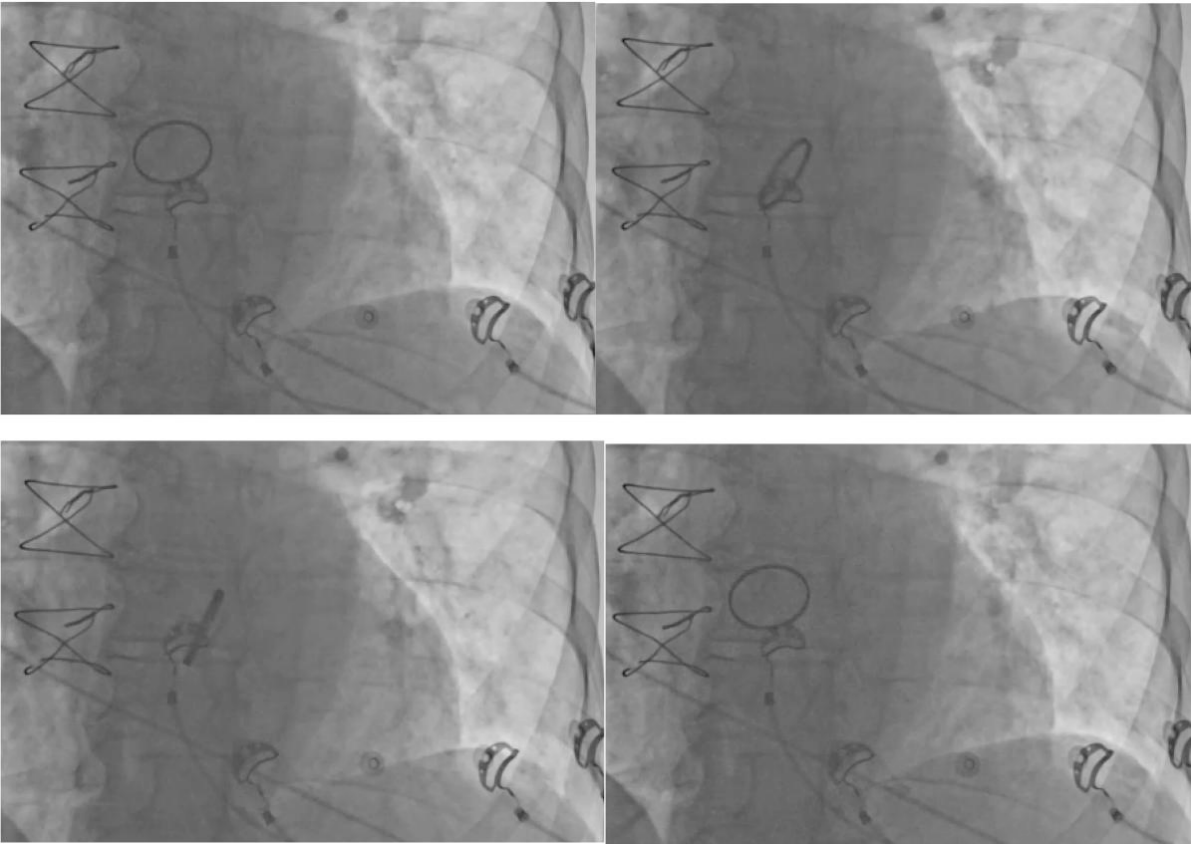
The rocking valve during intraoperative fluoroscopy for pacemaker implantation.

**Figure 2:**
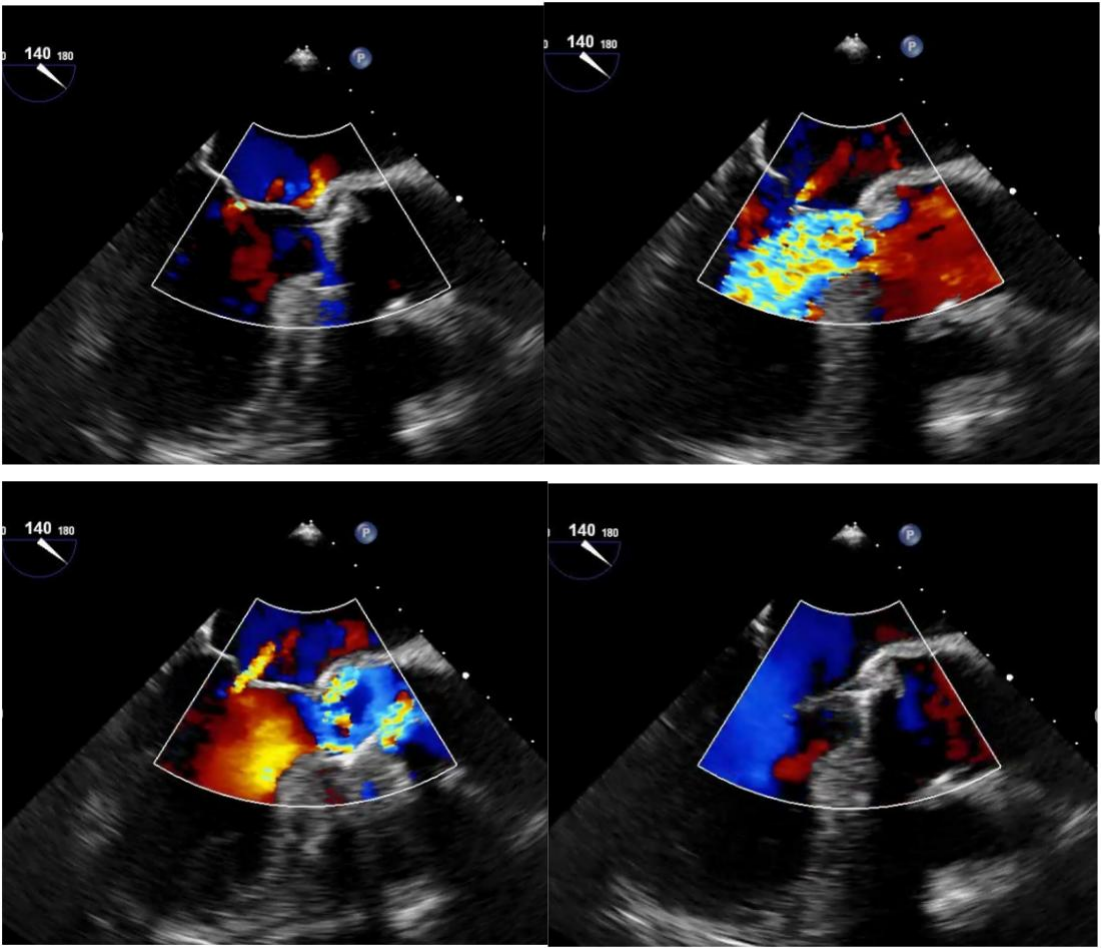
Transoesophageal echocardiogram mid-esophageal long-axis performed preoperatively.

During the procedure, intraoperative fluoroscopy incidentally revealed abnormal mobility of the mechanical aortic valve prosthesis, raising suspicion for prosthetic valve dehiscence (*Figure 1*). A transthoracic echocardiogram (TTE) confirmed this finding, demonstrating rocking motion of the prosthesis despite and no significant paravalvular regurgitation. Transoesophageal echocardiography (TOE) further delineated the extent of the detachment (*Figure 2*). Despite the absence of clinical signs of heart failure or infection (C-reactive protein and leucocytes), the imaging findings prompted an urgent surgical re-exploration.

**Figure 3:**
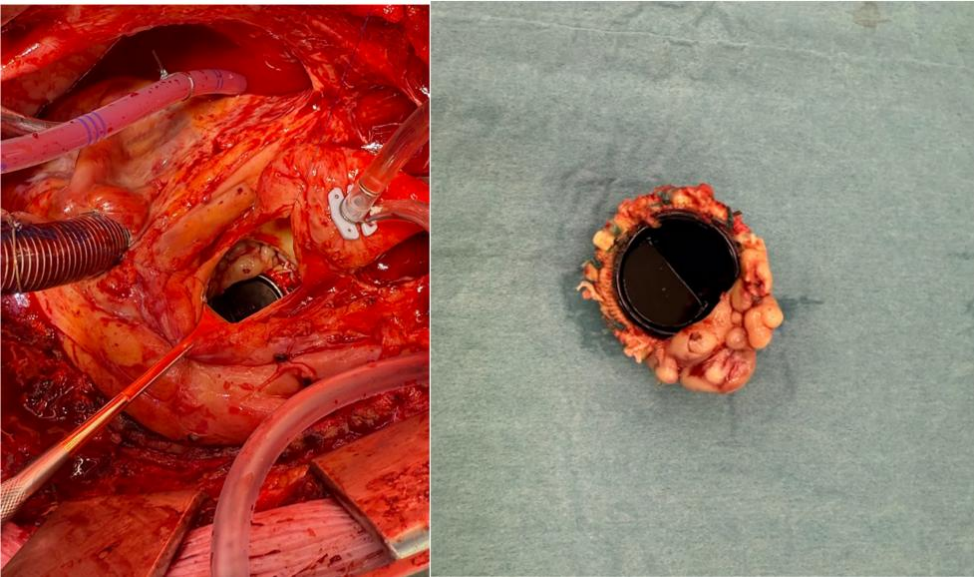
Intraoperative view of the mechanical valve and the vegetations seen through the aortotomy. The vegetation as seen around the resected mechanical valve.

Intraoperatively, the mechanical valve was found to be near circumferentially dehiscent from the aortic annulus (*Figure 3*). The explanted valve showed no mechanical dysfunction or thrombus but appeared surrounded by fibrotic and infectious tissue. No abscess cavity was identified.

The prosthesis was explanted and debridement was required, the mechanical prothesis was replaced with a 25 mm Carpentier-Edwards Perimount Magna Ease, in accordance with the patient’s wish for a bioprosthesis.

Both the aortic valve tissue cultures and multiple peripheral blood cultures taken prior to antibiotic treatment revealed Cutibacterium acnes, a low-virulence skin commensal known to cause indolent prosthetic valve endocarditis.^1^ Histopathological examination of surrounding tissue showed signs of chronic inflammation consistent with a low-grade infective process.

The postoperative course was uneventful. The patient recovered well and was discharged home in stable condition on targeted antibiotic therapy (intravenous Benzlypenicillin for a period of 6 weeks).^4^ At 6-week follow-up, he remained asymptomatic, with normal prosthetic function on follow-up TTE and no recurrence of conduction abnormalities beyond the original indication for pacemaker implantation.

## Discussion

This case describes a rare presentation of late prosthetic valve dehiscence caused by *Cutibacterium acnes*, detected incidentally in an asymptomatic patient. Although prosthetic valve dehiscence is a recognized complication of infective endocarditis, it is typically associated with acute infection and clear clinical manifestations. In contrast, this case demonstrates that low-grade infection may lead to extensive structural damage in the absence of systemic symptoms or haemodynamic compromise.

*Cutibacterium acnes* is an increasingly recognized cause of prosthetic valve endocarditis, characterized by an indolent clinical course, low inflammatory response, and diagnostic delay.^3^ The organism’s slow growth and its frequent classification as a contaminant may further obscure the diagnosis. In the present case, both blood and valve cultures confirmed *C. acnes*, supported by histopathological evidence of chronic inflammation, consistent with a low-grade infective process.

A notable aspect of this case is the absence of significant paravalvular regurgitation despite near circumferential dehiscence (>75%). This highlights that severe prosthetic dysfunction may remain clinically silent and underscores the importance of imaging in patients with prosthetic valves. The initial suspicion arose from fluoroscopy during an unrelated procedure, emphasizing the potential value of incidental findings in identifying otherwise occult but clinically relevant pathology.

This case underlines several important clinical messages. First, clinicians should consider indolent infective endocarditis in patients with prosthetic valves, even many years after implantation and in the absence of typical symptoms. Second, isolation of *C. acnes* should not be dismissed as contamination when there is supporting clinical or imaging evidence. Finally, prompt surgical intervention is warranted once significant prosthetic dehiscence is identified, given the risk of sudden deterioration.

In summary, case illustrates a rare, silent, and late presentation of mechanical valve dehiscence caused by *Cutibacterium acnes* infection. It underscores the importance of maintaining clinical vigilance and the potential role of incidental imaging in diagnosing life-threatening but clinically occult complications.

## Supplementary Material

ytag379_Supplementary_Data

## Data Availability

The data underlying this article are available in the article and its online Supplementary material. Further inquiries can be shared on reasonable request to the corresponding author.

## Lead author biography

Musa Nader is a resident in Catharina hospital in the department of cardiothoracic surgery in Eindhoven, The Netherlands.

## Notes

**Consent:** The authors confirm that written consent for submission and publication of this case report including image(s) and associated text has been obtained from the patient in line with COPE guidance.
